# Smokers with Multiple Chronic Disease Are More Likely to Quit Cigarette

**DOI:** 10.31586/gjeid.2024.1068

**Published:** 2024-10-30

**Authors:** Shervin Assari, Payam Sheikhattari

**Affiliations:** 1 Department of Internal Medicine, Charles R Drew University of Medicine and Science, Los Angeles, CA, USA; 2 Department of Urban Public Health, Charles R Drew University of Medicine and Science, Los Angeles, CA, USA; 3 Center for Urban Health Disparities Research and Innovation, Morgan State University, Baltimore, MD, USA; 4 The Prevention Sciences Research Center, School of Community Health and Policy, Morgan State University, Baltimore, MD, USA; 5 Department of Public and Allied Health, School of Community Health and Policy, Morgan State University, Baltimore, MD, USA

**Keywords:** Chronic Diseases, Tobacco Cessation, Tobacco Use, Public Health, Longitudinal Study

## Abstract

**Objective:**

This study aims to investigate the relationship between the presence of chronic medical conditions and cessation among U.S. adults who use combustible tobacco. We hypothesized that having chronic medical conditions would be associated with a higher likelihood of successfully quitting combustible tobacco.

**Methods:**

We utilized longitudinal data from the Population Assessment of Tobacco and Health (PATH) Study, using data from Waves 1 to 6. Only current daily smokers were included in our analysis. The independent variable was the number of chronic medical conditions, defined as zero, one, or two or more. The outcome was becoming a former smoker (quitting smoking). Using multivariate regression analyses, we assessed the association between the number of chronic conditions and tobacco cessation over the six waves. We controlled for potential confounding variables, including demographic factors and socioeconomic status.

**Results:**

Our analysis revealed a significant association between the number of chronic medical conditions and the likelihood of quitting smoking. Specifically, individuals with two or more chronic conditions exhibited a greater probability of quitting smoking compared to those with no chronic conditions. The results remained significant after adjusting for potential confounders.

**Conclusions:**

Multiple chronic medical conditions may act as a catalyst for smoking cessation among U.S. adults. This suggests that the presence of multimorbidity, defined as multiple chronic disease diagnoses, may serve as “teachable moments,” prompting significant health behavior changes. These findings highlight the potential for leveraging chronic disease management and healthcare interventions to promote tobacco cessation, particularly among individuals with multiple chronic conditions.

## 1. Introduction

Chronic diseases are increasingly prevalent in the United States [1], affecting millions of lives and influencing health behavior trends [2]. Among those with chronic conditions, tobacco use remains a major risk factor, contributing to numerous preventable comorbidities, exacerbations, complications, hospitalizations, and premature deaths [3].

Recent research has focused on understanding whether a diagnosis of a chronic medical condition serves as a catalyst for behavioral modification, specifically through the concept of the “teachable moment.” [4 5,6] This idea suggests that chronic disease diagnoses can prompt individuals to make significant changes in their health behaviors, such as quitting smoking [7,8].

The concept of a teachable moment is well-documented in studies examining changes in health behaviors following diagnoses of various chronic conditions, including cardiovascular diseases, diabetes, and cancer [9–12]. Research has shown that chronic disease diagnoses can lead to improved behaviors, such as reduced substance use, increased fruit and vegetable consumption, and increased physical activity [6,13]. However, the extent to which these diagnoses facilitate positive health behavior changes varies across different behaviors, and less is known about their impact on tobacco use.

While studies have shown mixed results regarding behavioral changes following receiving a diagnosis of chronic disease, research on smoking cessation following chronic disease diagnoses has been limited [3]. Observational studies have highlighted that individuals newly diagnosed with chronic conditions, including diabetes, may experience various changes in health behaviors, including smoking and alcohol consumption [7,8]. Some studies have reported beneficial behavior changes, while others suggest a lack of significant impact or even declines in behaviors such as physical activity [4,5,6].

Understanding whether chronic disease diagnoses can serve as a teachable moment for tobacco users is crucial [4,5,6]. Tobacco use is a leading cause of chronic diseases such as cardiovascular disease, respiratory disorders, and various cancers [3]. Therefore, identifying whether chronic disease diagnoses can trigger smoking cessation is of substantial public health interest [3]. Knowledge about whether chronic disease diagnoses lead to higher cessation rates could inform targeted interventions and policies aimed at reducing tobacco use and improving public health outcomes.

Additionally, the impact of a chronic disease diagnosis on smoking cessation may vary across different chronic disease, health behaviors, and subpopulations. Variations in resources, access to healthcare, and socioeconomic factors could influence how individuals respond to a diagnosis. Research suggests that individuals with limited resources or those living in communities with fewer opportunities for healthy behavior changes may face additional barriers to quitting smoking [14–19].

This paper aims to explore the relationship between chronic disease diagnoses at baseline and smoking cessation over time in the U.S. general adult population. By examining longitudinal data, this study seeks to test the hypothesis that having multiple chronic disease diagnoses may act as a teachable moment for quitting smoking, among adult smokers. Understanding these dynamics is critical for designing effective public health strategies and interventions to support tobacco cessation among adults with chronic medical conditions and ultimately reduce the burden of tobacco use in the U.S.

## 2. Methods

### 2.1. Study Design and Participants

This study employed longitudinal data from the Population Assessment of Tobacco and Health (PATH) Study [20,21], encompassing waves 1 through 6 with an 8-year follow-up period. The PATH Study is a nationally representative cohort study aimed at assessing tobacco use and its health impacts across a diverse adult population in the United States. For this analysis, we included participants who were adults at baseline (Wave 1) and had data available for all six waves to ensure an 8-year follow-up.

The study focused on individuals who were current smokers at baseline. Inclusion criteria required participants to be aged 18 years or older at Wave 1 and to have complete data across all six waves.

### 2.1. Variables and Measures

Chronic medical conditions were assessed at baseline through self-reports of conditions from a predefined list, including cardiovascular diseases (heart disease, congestive heart failure, heart attack, stroke), diabetes, chronic respiratory conditions (asthma, chronic bronchitis, emphysema, other lung disease), diabetes, gum disease, and cancer. Based on the number of conditions reported, participants were categorized into three groups: 0, 1, or 2 or more chronic conditions.

The primary outcome was smoking cessation, defined as self-reported abstinence from tobacco use for at least 6 months by Wave 6. Smoking status was evaluated at each wave, capturing details on the frequency and duration of abstinence.

Covariates included demographic factors such as age, sex, race/ethnicity, and educational attainment, as well as socioeconomic indicators including income level and employment status. Baseline smoking behavior was also considered, including the number of cigarettes smoked per day and smoking duration.

### 2.2. Statistical Analysis

Descriptive statistics were calculated to summarize the characteristics of the study sample, using means and standard deviations for continuous variables and frequencies and percentages for categorical variables. We conducted logistic regression analyses using Stata to examine the relationship between the number of chronic medical conditions and the likelihood of smoking cessation by Wave 6. Logistic regression models were performed with the number of chronic conditions (0, 1, or 2+) as a categorical predictor. All models were adjusted for potential confounders, including age, sex, race/ethnicity, education, income, employment status, and baseline smoking behavior. All statistical analyses were performed using Stata, with a significance level set at p < 0.05 for all tests.

We used survey design variables, including weights, strata, and primary sampling units (PSUs), in conjunction with the subpop option to focus specifically on daily smokers at baseline. This approach ensures that our findings are representative of the U.S. daily smoker population by accounting for the complex sampling design of the Population Assessment of Tobacco and Health (PATH) Study. By using these design variables, we adjusted for differential probabilities of selection, non-response, and post-stratification adjustments, thereby improving the generalizability of the results to the broader U.S. population of daily smokers. This methodology allows us to accurately reflect national estimates of smoking cessation behaviors and related factors among U.S. adults who were daily smokers at the study’s outset.

### 2.3. Ethical Considerations

The PATH Study received institutional review board approval from the coordinating centers, and all participants provided informed consent. Data used in this analysis were de-identified to maintain participant confidentiality.

## 3. Results

Table 1 presents the descriptive statistics for daily smokers at baseline who were followed for eight years to assess successful quitting. The age distribution showed that the largest group was individuals aged 25 to 34 years (24.3%, SE = 0.008), followed by those aged 45 to 54 years (22.1%, SE = 0.008) and 35 to 44 years (19.7%, SE = 0.008). The youngest age group (18 to 24 years) comprised 12.8% (SE = 0.006), while the smallest age groups were those aged 65 to 74 years (4.7%, SE = 0.005) and 75 years or older (0.4%, SE = 0.001).

The sample was nearly evenly split between males (50.7%, SE = 0.008) and females (49.3%, SE = 0.008). Regarding ethnicity, 90.2% (SE = 0.008) were non-Hispanic, and 9.8% (SE = 0.008) were Hispanic. The racial composition was predominantly White (75.5%, SE = 0.018), followed by Black individuals (17.7%, SE = 0.017) and individuals of other races (6.8%, SE = 0.006).

Geographically, most participants resided in the South (41.0%, SE = 0.019), followed by the Midwest (25.4%, SE = 0.018), the West (17.3%, SE = 0.015), and the Northeast (16.3%, SE = 0.017). Educational attainment varied, with the largest group having some college or an associate degree (34.3%, SE = 0.010), followed by high school graduates (28.6%, SE = 0.010), those with less than a high school education (16.5%, SE = 0.007), and GED holders (11.9%, SE = 0.007). Participants with a bachelor’s degree (6.9%, SE = 0.005) or an advanced degree (1.9%, SE = 0.003) were the least represented.

Regarding annual household income, 29.6% (SE = 0.010) of participants earned between $10,000 and $24,999, 24.8% (SE = 0.008) earned between $25,000 and $49,999, and 24.1% (SE = 0.010) earned less than $10,000. Only 16.0% (SE = 0.008) had an income between $50,000 and $99,999, and 5.4% (SE = 0.005) had an income of $100,000 or more.

Health insurance coverage varied, with 45.6% (SE = 0.010) having some private insurance, 25.4% (SE = 0.009) having no insurance, and 15.9% (SE = 0.008) having private insurance or Medicare only. Other insurance types were less common, including private insurance with some Medicare (9.7%, SE = 0.006) and other insurance only (3.3%, SE = 0.004).

Regarding chronic medical conditions, 47.3% (SE = 0.009) had no chronic conditions, 25.5% (SE = 0.009) had one chronic condition, and 27.3% (SE = 0.009) had two or more chronic conditions.

By the end of the eight-year follow-up, 81.3% (SE = 0.008) of the participants had successfully quit smoking, while 18.7% (SE = 0.008) continued smoking.

Table 2 summarizes the results of the survey logistic regression analysis that examined factors associated with the successful quit of smoking among daily smokers. Individuals with two or more chronic conditions had significantly higher odds of quitting smoking compared to those with no chronic conditions (OR: 1.32, 95% CI: 1.06, 1.64, p = 0.015). However, those with only one chronic condition did not show a statistically significant difference in the odds of quitting smoking compared to those with no chronic conditions (OR: 1.12, 95% CI: 0.90, 1.40, p = 0.295). The odds of quitting smoking were significantly lower across older age groups compared to the youngest age group (reference). For instance, compared to the youngest age group, individuals in age group 2 had 0.47 times the odds of quitting smoking (95% CI: 0.32, 0.70, p < 0.001), and the odds further decreased in older age groups, with age group 7 having the lowest odds (OR: 0.08, 95% CI: 0.02, 0.28, p < 0.001). Male sex was associated with higher odds of successfully quitting smoking compared to females (OR: 2.58, 95% CI: 2.14, 3.12, p < 0.001).

Hispanic ethnicity was not significantly associated with smoking cessation compared to non-Hispanic individuals (OR: 0.91, 95% CI: 0.63, 1.32, p = 0.633). Regarding race, Black individuals had slightly lower odds of quitting smoking compared to White individuals, but the difference was not statistically significant (OR: 0.86, 95% CI: 0.64, 1.14, p = 0.289). The odds of quitting was not different for individuals of other races from White individuals (OR: 1.08, 95% CI: 0.71, 1.65, p = 0.722).

No significant differences were found across census regions, with all regions showing similar odds of smoking cessation compared to the reference region (Northeast). Education level was not significantly associated with smoking cessation, as the odds ratios for different education levels were close to 1 and not statistically significant. Annual household income did not show a consistent pattern of association with smoking cessation, with odds ratios near 1 across income categories, none of which were statistically significant. Having health insurance was not significantly associated with quitting smoking (OR: 0.98, 95% CI: 0.92, 1.04, p = 0.474). These results suggest that age, sex, and having multiple chronic medical conditions are key factors associated with successful smoking cessation among daily smokers.

## 4. Discussion

The aim of this study was to investigate the relationship between the presence of chronic medical conditions and the likelihood of smoking cessation among U.S. adults. Specifically, we sought to determine whether having multiple chronic conditions is associated with a higher probability of quitting smoking over an 8-year follow-up period.

Our analysis of data from the PATH Study [20,21] revealed a significant association between the number of chronic medical conditions and the likelihood of smoking cessation. Participants with a higher number of chronic conditions were more likely to quit smoking compared to those with fewer or no chronic conditions. This finding underscores the potential role of chronic disease diagnoses as a catalyst for tobacco cessation.

Several factors may contribute to why individuals with chronic medical conditions are more likely to quit smoking. Chronic diseases often come with increased health risks and complications, which may heighten the perceived urgency for improving health behaviors. The diagnosis of a chronic condition may act as a strong motivator, prompting individuals to reassess their health priorities and make significant lifestyle changes. For some, the diagnosis may trigger a renewed focus on health and well-being, making smoking cessation a more immediate and compelling goal.

The decision to quit smoking among individuals with chronic conditions may be influenced by both fear and motivation. Fear of worsening health or facing additional complications from smoking may drive individuals to quit, while motivation to improve health outcomes and enhance quality of life can reinforce this decision. The interaction between these factors is complex, and both fear and motivation likely play a role in encouraging smoking cessation.

Despite these insights, several aspects remain unclear. We do not fully understand the role of external support in the cessation process, such as the influence of healthcare providers and social support networks. Additionally, it is not clear which specific chronic conditions are most strongly associated with smoking cessation or whether certain types of smoking cessation products (e.g., nicotine replacement therapy, prescription medications) are more effective for individuals with chronic conditions. Understanding these nuances could provide a more comprehensive picture of the cessation process for this population.

### 4.1. Limitations

This study has a few limitations. First, the reliance on self-reported data for smoking status and chronic conditions may introduce reporting biases. Second, while the PATH Study provides a rich dataset, it does not capture all potential factors influencing smoking cessation, such as mental health status or detailed information on cessation support. Additionally, the observational nature of the study limits our ability to establish causality. Finally, the heterogeneity within the category of “chronic conditions” may obscure specific condition-related effects on smoking cessation.

### 4.2. Future Research

Future research should address these gaps by exploring the role of healthcare provider support and social networks in smoking cessation among individuals with chronic conditions. Studies should also investigate the effectiveness of different smoking cessation products within this population and examine the impact of specific chronic conditions on cessation rates. Longitudinal studies with more detailed data on cessation support and mental health could provide further insights into the factors influencing smoking cessation. Future qualitative studies that capture the nature of teachable moments and the driving factors leading to successful quit attempts can shed light and generate important hypotheses on the determinants of success. These studies could also identify ways to apply these teachable moments to individuals who have not yet developed chronic illnesses. Additionally, studying the potential effectiveness of interventions aimed at disseminating knowledge that contributes to the success of people with chronic illnesses could help motivate those who have not yet developed such conditions.

### 4.3. Implications

The findings of this study have several public health implications. Given that individuals with chronic conditions are more likely to quit smoking, healthcare providers should leverage chronic disease diagnoses as teachable moments to promote tobacco cessation. Targeted interventions that address the unique needs and barriers faced by this population could enhance smoking cessation efforts. Public health policies should also focus on providing accessible cessation resources and support for individuals with chronic conditions.

## 5. Conclusions

In conclusion, our study demonstrates that the presence of multiple chronic medical conditions is associated with an increased likelihood of smoking cessation among U.S. adults. While the chronic disease diagnosis may act as a significant motivator for quitting, a more nuanced understanding of the support mechanisms and specific conditions involved is needed. Addressing these factors through tailored interventions and comprehensive support strategies could improve smoking cessation outcomes and contribute to better overall health for individuals with chronic conditions.

## Figures and Tables

**Table 1.** Descriptive Data (Weighted in Daily Smokers at Time One who were followed for successful quitting for eight years)

	%	SE	95%	CI
Age				
18 to 24 years old	12.811	0.006	11.721	13.986
25 to 34 years old	24.269	0.008	22.623	25.995
35 to 44 years old	19.680	0.008	18.167	21.286
45 to 54 years old	22.071	0.008	20.556	23.664
55 to 64 years old	16.069	0.008	14.554	17.709
65 to 74 years old	4.665	0.005	3.828	5.675
75 years old or older	0.435	0.001	0.223	0.847
Sex				
Female	49.257	0.008	47.580	50.936
Male	50.743	0.008	49.064	52.420
Ethnicity				
Non-Hispanic	90.202	0.008	88.542	91.643
Hispanic	9.798	0.008	8.357	11.458
Race				
White	75.541	0.018	71.901	78.849
Black	17.709	0.017	14.584	21.337
Other	6.750	0.006	5.713	7.959
Census Region				
Northeast	16.329	0.017	13.306	19.882
Midwest	25.397	0.018	22.019	29.100
South	40.963	0.019	37.322	44.707
West	17.310	0.015	14.606	20.395
Education				
Less than high school	16.533	0.007	15.148	18.018
GED	11.866	0.007	10.624	13.233
High school graduate	28.554	0.010	26.575	30.619
Some college (no degree) or associate degree	34.263	0.010	32.273	36.310
Bachelor’s degree	6.876	0.005	5.934	7.955
Advanced degree	1.907	0.003	1.457	2.493
Annual Household Income				
Less than $10,000	24.137	0.010	22.276	26.102
$10,000 to $24,999	29.588	0.010	27.735	31.510
$25,000 to $49,999	24.823	0.008	23.232	26.486
$50,000 to $99,999	16.045	0.008	14.579	17.629
$100,000 or more	5.407	0.005	4.525	6.448
Health Insurance				
Some private insurance	45.626	0.010	43.664	47.603
Private insurance, some Medicare	9.707	0.006	8.636	10.894
Private insurance or Medicare, s	15.982	0.008	14.439	17.657
Other insurance only	3.328	0.004	2.653	4.167
No insurance	25.357	0.009	23.538	27.266
Chronic Medical Conditions				
0	47.281	0.009	45.531	49.038
1	25.460	0.009	23.799	27.195
2+	27.258	0.009	25.563	29.022
Successful Quitting (by Year 8)				
No	18.656	0.008	17.081	20.340
Yes	81.344	0.008	79.660	82.919

**Table 2.** Summary of survey logistic regression: Outcome: Successful Quit of Smoking among Daily Smokers)

	Odds Ratio	Linearized std. err.	95%	CI	p
Age					
18 to 24 years old	Ref				
25 to 34 years old	0.47	0.09	0.32	0.70	< 0.001
35 to 44 years old	0.36	0.08	0.23	0.57	< 0.001
45 to 54 years old	0.21	0.04	0.14	0.31	< 0.001
55 to 64 years old	0.18	0.04	0.12	0.28	< 0.001
65 to 74 years old	0.16	0.05	0.09	0.29	< 0.001
75 years old or older	0.08	0.05	0.02	0.28	< 0.001
Male Sex	2.58	0.24	2.14	3.12	< 0.001
Hispanic Ethnicity	0.91	0.17	0.63	1.32	0.633
Race					
White	Ref.				
Black	0.86	0.12	0.64	1.14	0.289
Other	1.08	0.23	0.71	1.65	0.722
Census Region					
Northeast	Ref.				
Midwest	1.23	0.20	0.88	1.70	0.223
South	1.12	0.17	0.82	1.52	0.467
West	1.26	0.23	0.87	1.81	0.215
Education Level					
Less than high school	Ref.				
GED	0.76	0.14	0.52	1.09	0.133
High school graduate	0.83	0.12	0.63	1.11	0.204
Some college (no degree) or associate	1.00	0.14	0.76	1.31	0.997
Bachelor’s degree or	0.82	0.21	0.50	1.36	0.434
Advanced degree	1.06	0.40	0.49	2.25	0.888
Annual Household Income					
Less than $10,000	Ref.				
$10,000 to $24,999	1.05	0.14	0.80	1.36	0.734
$25,000 to $49,999	1.03	0.16	0.76	1.40	0.824
$50,000 to $99,999	0.87	0.14	0.63	1.20	0.380
$100,000 or more	1.34	0.37	0.77	2.33	0.299
Health Insurance (Any)	0.98	0.03	0.92	1.04	0.474
Chronic Medical Conditions					
0	Ref.				
1	1.12	0.12	0.90	1.40	0.295
2	1.32	0.15	1.06	1.64	0.015
Intercept	8.86	2.01	5.65	13.88	< 0.001
